# Ethyl 4,9-dimethyl-9*H*-carbazole-3-carboxyl­ate

**DOI:** 10.1107/S1600536814002098

**Published:** 2014-02-05

**Authors:** Serkan Öncüoğlu, Nefise Dilek, Nagihan Çaylak Delibaş, Yavuz Ergün, Tuncer Hökelek

**Affiliations:** a Dokuz Eylül University, Faculty of Arts and Sciences, Department of Chemistry, Tınaztepe, 35160 Buca, İzmir, Turkey; b Aksaray University, Department of Physics, 68100, Aksaray, Turkey; c Department of Physics, Sakarya University, 54187 Esentepe, Sakarya, Turkey; d Hacettepe University, Department of Physics, 06800 Beytepe, Ankara, Turkey

## Abstract

In the title compound, C_17_H_17_NO_2_, the carbazole skeleton includes an eth­oxy­carbonyl group at the 3-position. The indole three-ring system is almost planar [maximum deviation = 0.065 (2) Å], and the ethyl ester group is inclined to its mean plane by 15.48 (2)°. In the crystal, there are π–π stacking inter­actions between parallel benzene rings and between parallel benzene and pyrrole rings of adjacent mol­ecules [centroid–centroid distances = 3.9473 (8) and 3.7758 (8) Å, respectively]. Weak C—H⋯π inter­actions are also present.

## Related literature   

For the first isolation of carbazole from coal tar, see: Graebe & Glazer (1872 ▶). For the isolation of murrayanine, the first report of a naturally occurring carbazole alkaloid, see: Chakraborty *et al.* (1965 ▶). For the intriguing structural features and promising biological activities exhibited by many carbazole alkaloids, see: Chakraborty (1993 ▶). For the syntheses of pyridocarbazoles, see: Karmakar *et al.* (1991 ▶). For related structures, see: Hökelek *et al.* (1994 ▶); Patır *et al.* (1997 ▶). For bond-length data, see: Allen *et al.* (1987 ▶).
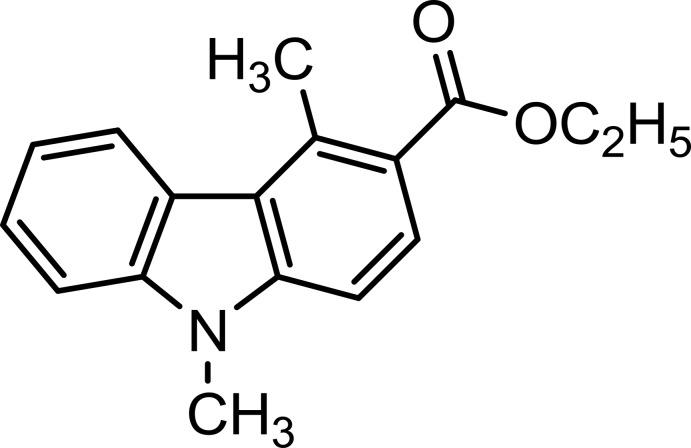


## Experimental   

### 

#### Crystal data  

C_17_H_17_NO_2_
*M* *_r_* = 267.32
Orthorhombic, 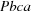

*a* = 14.5228 (5) Å
*b* = 12.4663 (4) Å
*c* = 15.2354 (5) Å
*V* = 2758.30 (16) Å^3^
*Z* = 8
Mo *K*α radiation
μ = 0.08 mm^−1^
*T* = 296 K
0.43 × 0.35 × 0.25 mm

#### Data collection  

Bruker SMART BREEZE CCD diffractometer
Absorption correction: multi-scan (*SADABS*; Bruker, 2007 ▶) *T*_min_ = 0.965, *T*_max_ = 0.979
61052 measured reflections
2798 independent reflections
2358 reflections with *I* > 2σ(*I*)
*R*_int_ = 0.041

#### Refinement  

*R*[*F* ^2^ > 2σ(*F* ^2^)] = 0.049
*wR*(*F* ^2^) = 0.133
*S* = 1.07
2798 reflections
185 parameters
H-atom parameters constrained
Δρ_max_ = 0.23 e Å^−3^
Δρ_min_ = −0.18 e Å^−3^

### 

Data collection: *APEX2* (Bruker, 2007 ▶); cell refinement: *SAINT* (Bruker, 2007 ▶); data reduction: *SAINT*; program(s) used to solve structure: *SHELXS97* (Sheldrick, 2008 ▶); program(s) used to refine structure: *SHELXL97* (Sheldrick, 2008 ▶); molecular graphics: *ORTEP-3* for Windows (Farrugia, 2012 ▶); software used to prepare material for publication: *WinGX* (Farrugia, 2012 ▶) and *PLATON* (Spek, 2009 ▶).

## Supplementary Material

Crystal structure: contains datablock(s) I, global. DOI: 10.1107/S1600536814002098/su2693sup1.cif

Structure factors: contains datablock(s) I. DOI: 10.1107/S1600536814002098/su2693Isup2.hkl

Supporting information file. DOI: 10.1107/S1600536814002098/su2693Isup3.cml

CCDC reference:

Additional supporting information:  crystallographic information; 3D view; checkCIF report

## Figures and Tables

**Table 1:** Hydrogen-bond geometry (Å, °) *Cg*1, *Cg*2 and *Cg*3 are the centroids of rings N9/C8*A*/C5*A*/C4*A*/C9*A*, C1–C4/C4*A*/C9*A*, and C5/C5*A*/C8*A*/C8/C7/C6, respectively.

*D*—H⋯*A*	*D*—H	H⋯*A*	*D*⋯*A*	*D*—H⋯*A*
C8—H8⋯*Cg*1^i^	0.93	2.83	3.7091 (17)	159
C13—H13*A*⋯*Cg*2^ii^	0.97	2.91	3.6381 (17)	133
C14—H14*C*⋯*Cg*3^ii^	0.96	2.73	3.580 (2)	149

Symmetry codes: (i) 
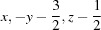
; (ii) 
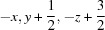
.

**Fig. 1.**
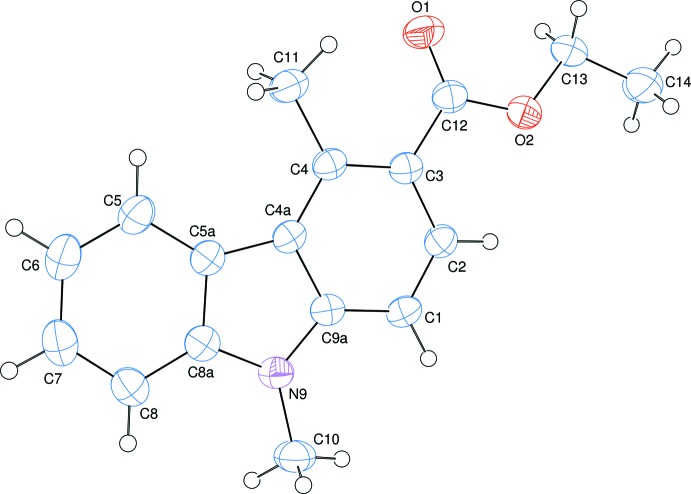
The molecular structure of the title molecule, with atom labelling. Displacement ellipsoids are drawn at the 50% probability level.

**Fig. 2.**
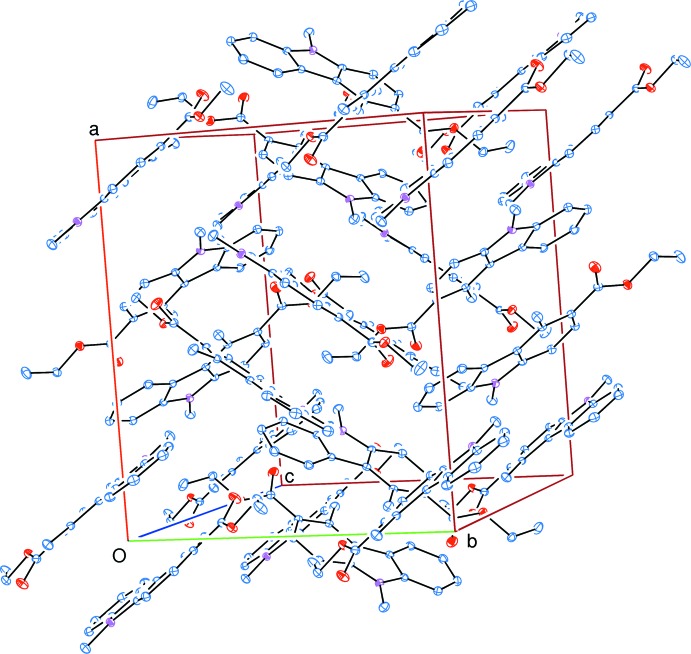
A view of the crystal packing of the title compound [H-atoms have been omitted for clarity].

## supplementary crystallographic information

### 1. Comment

Carbazole was firstly isolated from coal tar many years ago (Graebe & Glazer, 1872). The isolation of murrayanine was the first report of a naturally occurring carbazole alkaloid (Chakraborty *et al.*, 1965). Since then there has been a strong interest in this area by chemists and biologists due to the intriguing structural features and promising biological activities exhibited by many carbazole alkaloids (Chakraborty, 1993). Most carbazole alkaloids have been isolated from the taxonomically related higher plants of the genus *Murraya*, *Glycosmis* and *Clausena* from the family Rutaceae. The genus *Murraya* represents the richest source of carbazole alkaloids from terrestrial plants. The title compound was used as a precursor compound for the syntheses of pyridocarbazoles (Karmakar *et al.*, 1991) and we report herein on its crystal structure.

The molecule of the title compound, Fig. 1, contains a carbazole skeleton with an ethoxycarbonyl group at the 3 position. The bond lengths are close to standard values (Allen *et al.*, 1987) and generally agree with those in previously reported compounds (Hökelek *et al.*, 1994; Patır *et al.*, 1997). In all structures atom N9 is substituted.

An examination of the deviations from the least-squares planes through individual rings shows that rings *A* (C1—C4/C4a/c9a), *B* (C4a/C5a/C8a/N9/C9a) and *C* (C5a/C5—C8/C8a) are nearly coplanar [with a maximum deviation of 0.065 (2) Å for atom C7] with dihedral angles of A/B = 2.41 (4) °, A/C = 4.01 (4) ° and B/C = 1.63 (5) °. Atoms C10, C11 and C12 are displaced by -0.059 (2), -0.092 (2) and 0.079 (2) Å from the adjacent ring planes.

In the crystal, π–π contacts between the benzene rings and between the benzene and pyrrole rings, Cg1···Cg1^i^ and Cg2···Cg1^i^ [symmetry code: (i) 1 - *x*, - *y*, 1 - *z*, where Cg1 and Cg2 are the centroids of the rings *A* (C1—C4/C4a/C9a) and *B* (C4a/C5a/C8a/N9/C9a), respectively] stabilize the crystal structure, with centroid-centroid distances of 3.9473 (8) and 3.7758 (8) Å. The weak C—H···π interactions (Table 1) may be further effective in the stabilization of the crystal structure.

### 2. Experimental

A solution of ethyl 4-methyl-9*H*-carbazole-3-carboxylate (2.50 g, 10 mmol), potassium hydroxide (1.70 g, 30 mmol) and methyl iodide (1.25 ml, 20 mmol) in acetone (100 ml) was stirred at 273 K for 2 h, and then acidified with HCl (6N). The aqueous layer was extracted with dichloromethane. The combined organic phases were dried with magnesium sulfate, filtered, and the solvents were evaporated. The residue was purified by column chromatography using silica gel, hexane/ethyl acetate (1:1). After the solvent was evaporated, the crude product was recrystallized from methanol (yield 87%, M.p. 383 K), giving colourless prismatic crystals.

### 3. Refinement

The C-bound H-atoms were positioned geometrically with C—H = 0.93, 0.97 and 0.96 Å, for aromatic, methylene and methyl H-atoms, respectively, and constrained to ride on their parent atoms, with *U*_iso_(H) = k × *U*_eq_(C), where k = 1.5 for methyl H-atoms and = 1.2 for other H-atoms.

### Crystal data

**Table:**

C_17_H_17_NO_2_	*F*(000) = 1136
*M**_r_* = 267.32	*D*_x_ = 1.287 Mg m^−^^3^
Orthorhombic, *P**b**c**a*	Mo *K*α radiation, λ = 0.71073 Å
Hall symbol: -P 2ac 2ab	Cell parameters from 9733 reflections
*a* = 14.5228 (5) Å	θ = 2.5–28.6°
*b* = 12.4663 (4) Å	µ = 0.08 mm^−^^1^
*c* = 15.2354 (5) Å	*T* = 296 K
*V* = 2758.30 (16) Å^3^	Prism, colourless
*Z* = 8	0.43 × 0.35 × 0.25 mm

### Data collection

**Table:**

Bruker SMART BREEZE CCD diffractometer	2798 independent reflections
Radiation source: fine-focus sealed tube	2358 reflections with *I* > 2σ(*I*)
Graphite monochromator	*R*_int_ = 0.041
φ and ω scans	θ_max_ = 26.4°, θ_min_ = 2.5°
Absorption correction: multi-scan (*SADABS*; Bruker, 2007)	*h* = −18→18
*T*_min_ = 0.965, *T*_max_ = 0.979	*k* = −15→15
61052 measured reflections	*l* = −19→19

### Refinement

**Table:**

Refinement on *F*^2^	Secondary atom site location: difference Fourier map
Least-squares matrix: full	Hydrogen site location: inferred from neighbouring sites
*R*[*F*^2^ > 2σ(*F*^2^)] = 0.049	H-atom parameters constrained
*wR*(*F*^2^) = 0.133	*w* = 1/[σ^2^(*F*_o_^2^) + (0.0716*P*)^2^ + 0.6943*P*] where *P* = (*F*_o_^2^ + 2*F*_c_^2^)/3
*S* = 1.07	(Δ/σ)_max_ < 0.001
2798 reflections	Δρ_max_ = 0.23 e Å^−^^3^
185 parameters	Δρ_min_ = −0.18 e Å^−^^3^
0 restraints	Extinction correction: *SHELXL97* (Sheldrick, 2008), Fc^*^=kFc[1+0.001xFc^2^λ^3^/sin(2θ)]^-1/4^
Primary atom site location: structure-invariant direct methods	Extinction coefficient: 0.0071 (10)

### Special details

**Table:**

Geometry. All esds (except the esd in the dihedral angle between two l.s. planes) are estimated using the full covariance matrix. The cell esds are taken into account individually in the estimation of esds in distances, angles and torsion angles; correlations between esds in cell parameters are only used when they are defined by crystal symmetry. An approximate (isotropic) treatment of cell esds is used for estimating esds involving l.s. planes.
Refinement. Refinement of F^2^ against ALL reflections. The weighted R-factor wR and goodness of fit S are based on F^2^, conventional R-factors R are based on F, with F set to zero for negative F^2^. The threshold expression of F^2^ > 2sigma(F^2^) is used only for calculating R-factors(gt) etc. and is not relevant to the choice of reflections for refinement. R-factors based on F^2^ are statistically about twice as large as those based on F, and R- factors based on ALL data will be even larger.

### Fractional atomic coordinates and isotropic or equivalent isotropic displacement parameters (Å^2^)

**Table:**

	*x*	*y*	*z*	*U*_iso_*/*U*_eq_	
O1	0.41026 (9)	0.81153 (11)	0.06716 (8)	0.0672 (4)	
O2	0.46532 (8)	0.86264 (9)	−0.06240 (7)	0.0529 (3)	
C1	0.62278 (10)	0.60151 (12)	−0.10399 (9)	0.0442 (4)	
H1	0.6469	0.5902	−0.1598	0.053*	
C2	0.56446 (10)	0.68566 (12)	−0.08727 (9)	0.0430 (3)	
H2	0.5484	0.7312	−0.1331	0.052*	
C3	0.52798 (9)	0.70562 (11)	−0.00294 (9)	0.0396 (3)	
C4	0.55188 (9)	0.63966 (12)	0.06812 (9)	0.0392 (3)	
C4A	0.60974 (9)	0.55202 (11)	0.05131 (8)	0.0376 (3)	
C5	0.63676 (11)	0.43336 (14)	0.19289 (10)	0.0503 (4)	
H5	0.6028	0.4749	0.2320	0.060*	
C5A	0.64528 (9)	0.46499 (12)	0.10518 (9)	0.0404 (3)	
C6	0.67876 (12)	0.34056 (15)	0.22115 (11)	0.0585 (4)	
H6	0.6730	0.3200	0.2796	0.070*	
C7	0.72949 (12)	0.27711 (15)	0.16397 (12)	0.0585 (4)	
H7	0.7568	0.2146	0.1847	0.070*	
C8	0.74011 (11)	0.30520 (13)	0.07687 (11)	0.0507 (4)	
H8	0.7744	0.2630	0.0386	0.061*	
C8A	0.69762 (9)	0.39910 (12)	0.04852 (10)	0.0419 (3)	
N9	0.69734 (8)	0.44270 (10)	−0.03478 (8)	0.0430 (3)	
C9A	0.64444 (9)	0.53372 (11)	−0.03426 (9)	0.0392 (3)	
C10	0.74717 (13)	0.40054 (15)	−0.10959 (11)	0.0575 (4)	
H10A	0.7397	0.3241	−0.1120	0.086*	
H10B	0.8114	0.4176	−0.1039	0.086*	
H10C	0.7235	0.4320	−0.1625	0.086*	
C11	0.52039 (12)	0.66026 (15)	0.16070 (10)	0.0564 (4)	
H11A	0.5690	0.6424	0.2008	0.085*	
H11B	0.4674	0.6168	0.1732	0.085*	
H11C	0.5047	0.7346	0.1672	0.085*	
C12	0.46242 (10)	0.79659 (12)	0.00684 (9)	0.0441 (3)	
C13	0.39819 (11)	0.94836 (13)	−0.06466 (11)	0.0515 (4)	
H13A	0.3366	0.9200	−0.0567	0.062*	
H13B	0.4104	0.9999	−0.0184	0.062*	
C14	0.40701 (16)	0.99994 (19)	−0.15226 (13)	0.0778 (6)	
H14A	0.3947	0.9480	−0.1973	0.117*	
H14B	0.4684	1.0273	−0.1592	0.117*	
H14C	0.3637	1.0578	−0.1568	0.117*	

### Atomic displacement parameters (Å^2^)

**Table:**

	*U*^11^	*U*^22^	*U*^33^	*U*^12^	*U*^13^	*U*^23^
O1	0.0694 (8)	0.0763 (9)	0.0559 (7)	0.0237 (7)	0.0186 (6)	0.0033 (6)
O2	0.0516 (6)	0.0491 (7)	0.0579 (7)	0.0120 (5)	0.0104 (5)	0.0057 (5)
C1	0.0473 (8)	0.0509 (9)	0.0345 (7)	0.0007 (7)	0.0044 (5)	−0.0033 (6)
C2	0.0446 (7)	0.0460 (8)	0.0383 (7)	−0.0001 (6)	−0.0004 (6)	0.0006 (6)
C3	0.0356 (7)	0.0423 (8)	0.0408 (7)	−0.0030 (6)	0.0010 (5)	−0.0033 (6)
C4	0.0352 (6)	0.0435 (8)	0.0390 (7)	−0.0055 (6)	0.0030 (5)	−0.0036 (6)
C4A	0.0334 (6)	0.0419 (8)	0.0374 (7)	−0.0071 (6)	0.0005 (5)	−0.0025 (5)
C5	0.0458 (8)	0.0594 (10)	0.0458 (8)	−0.0044 (7)	0.0038 (6)	0.0060 (7)
C5A	0.0338 (6)	0.0436 (8)	0.0437 (7)	−0.0063 (6)	−0.0006 (5)	0.0001 (6)
C6	0.0559 (9)	0.0665 (11)	0.0532 (9)	−0.0073 (8)	−0.0007 (7)	0.0171 (8)
C7	0.0534 (9)	0.0508 (10)	0.0712 (11)	−0.0027 (8)	−0.0062 (8)	0.0153 (8)
C8	0.0441 (8)	0.0451 (9)	0.0628 (9)	−0.0004 (6)	−0.0019 (7)	0.0011 (7)
C8A	0.0355 (7)	0.0426 (8)	0.0476 (7)	−0.0064 (6)	−0.0019 (5)	−0.0014 (6)
N9	0.0436 (7)	0.0442 (7)	0.0413 (6)	0.0027 (5)	0.0027 (5)	−0.0040 (5)
C9A	0.0358 (7)	0.0416 (8)	0.0401 (7)	−0.0031 (6)	0.0009 (5)	−0.0049 (6)
C10	0.0607 (10)	0.0618 (11)	0.0501 (9)	0.0137 (8)	0.0077 (7)	−0.0093 (8)
C11	0.0616 (10)	0.0654 (11)	0.0422 (8)	0.0109 (8)	0.0111 (7)	−0.0006 (7)
C12	0.0407 (7)	0.0477 (8)	0.0441 (7)	−0.0008 (6)	0.0009 (6)	−0.0034 (6)
C13	0.0504 (9)	0.0475 (9)	0.0567 (9)	0.0118 (7)	0.0059 (7)	−0.0034 (7)
C14	0.0905 (15)	0.0754 (14)	0.0675 (12)	0.0381 (12)	0.0201 (10)	0.0156 (10)

### Geometric parameters (Å, º)

**Table:**

O1—C12	1.2054 (18)	C8—C7	1.381 (2)
O2—C12	1.3388 (18)	C8—H8	0.9300
O2—C13	1.4469 (18)	C8A—C8	1.392 (2)
C1—H1	0.9300	N9—C8A	1.3805 (19)
C2—C1	1.372 (2)	N9—C9A	1.3704 (18)
C2—H2	0.9300	N9—C10	1.4488 (19)
C3—C2	1.4119 (19)	C9A—C1	1.393 (2)
C3—C12	1.488 (2)	C10—H10A	0.9600
C4—C3	1.403 (2)	C10—H10B	0.9600
C4—C11	1.5049 (19)	C10—H10C	0.9600
C4A—C4	1.402 (2)	C11—H11A	0.9600
C4A—C5A	1.455 (2)	C11—H11B	0.9600
C4A—C9A	1.4162 (18)	C11—H11C	0.9600
C5—C6	1.377 (2)	C13—C14	1.487 (2)
C5—H5	0.9300	C13—H13A	0.9700
C5A—C5	1.399 (2)	C13—H13B	0.9700
C5A—C8A	1.413 (2)	C14—H14A	0.9600
C6—C7	1.388 (3)	C14—H14B	0.9600
C6—H6	0.9300	C14—H14C	0.9600
C7—H7	0.9300		
			
C12—O2—C13	116.86 (12)	C8A—N9—C10	125.37 (13)
C2—C1—C9A	117.48 (13)	C9A—N9—C8A	108.81 (11)
C2—C1—H1	121.3	C9A—N9—C10	125.80 (13)
C9A—C1—H1	121.3	N9—C9A—C1	128.64 (12)
C1—C2—C3	122.36 (13)	N9—C9A—C4A	109.76 (12)
C1—C2—H2	118.8	C1—C9A—C4A	121.59 (13)
C3—C2—H2	118.8	N9—C10—H10A	109.5
C2—C3—C12	117.74 (13)	N9—C10—H10B	109.5
C4—C3—C2	120.40 (13)	N9—C10—H10C	109.5
C4—C3—C12	121.85 (12)	H10A—C10—H10B	109.5
C3—C4—C11	123.23 (13)	H10C—C10—H10A	109.5
C4A—C4—C3	117.65 (12)	H10C—C10—H10B	109.5
C4A—C4—C11	119.11 (13)	C4—C11—H11A	109.5
C4—C4A—C5A	133.68 (12)	C4—C11—H11B	109.5
C4—C4A—C9A	120.47 (13)	C4—C11—H11C	109.5
C9A—C4A—C5A	105.84 (12)	H11B—C11—H11A	109.5
C5A—C5—H5	120.1	H11B—C11—H11C	109.5
C6—C5—C5A	119.77 (15)	H11C—C11—H11A	109.5
C6—C5—H5	120.1	O1—C12—O2	121.70 (14)
C5—C5A—C4A	135.87 (14)	O1—C12—C3	126.58 (14)
C5—C5A—C8A	117.86 (14)	O2—C12—C3	111.69 (12)
C8A—C5A—C4A	106.23 (12)	O2—C13—C14	106.43 (13)
C5—C6—C7	121.17 (15)	O2—C13—H13A	110.4
C5—C6—H6	119.4	O2—C13—H13B	110.4
C7—C6—H6	119.4	C14—C13—H13A	110.4
C8—C7—C6	121.18 (16)	C14—C13—H13B	110.4
C8—C7—H7	119.4	H13B—C13—H13A	108.6
C6—C7—H7	119.4	C13—C14—H14A	109.5
C7—C8—C8A	117.51 (16)	C13—C14—H14B	109.5
C7—C8—H8	121.2	C13—C14—H14C	109.5
C8A—C8—H8	121.2	H14B—C14—H14A	109.5
N9—C8A—C5A	109.34 (13)	H14B—C14—H14C	109.5
N9—C8A—C8	128.14 (14)	H14C—C14—H14A	109.5
C8—C8A—C5A	122.52 (14)		
			
C13—O2—C12—O1	−4.5 (2)	C5A—C4A—C9A—N9	0.13 (15)
C13—O2—C12—C3	174.05 (12)	C5A—C4A—C9A—C1	178.99 (13)
C12—O2—C13—C14	−172.34 (16)	C5A—C5—C6—C7	−0.2 (2)
C3—C2—C1—C9A	1.0 (2)	C4A—C5A—C5—C6	177.19 (15)
C4—C3—C2—C1	1.0 (2)	C8A—C5A—C5—C6	0.0 (2)
C12—C3—C2—C1	−177.59 (13)	C4A—C5A—C8A—N9	1.47 (15)
C2—C3—C12—O1	162.94 (16)	C4A—C5A—C8A—C8	−177.90 (13)
C2—C3—C12—O2	−15.50 (18)	C5—C5A—C8A—N9	179.47 (13)
C4—C3—C12—O1	−15.7 (2)	C5—C5A—C8A—C8	0.1 (2)
C4—C3—C12—O2	165.90 (13)	C5—C6—C7—C8	0.4 (3)
C4A—C4—C3—C2	−2.4 (2)	C8A—C8—C7—C6	−0.4 (2)
C4A—C4—C3—C12	176.15 (12)	N9—C8A—C8—C7	−179.14 (15)
C11—C4—C3—C2	176.05 (14)	C5A—C8A—C8—C7	0.1 (2)
C11—C4—C3—C12	−5.4 (2)	C9A—N9—C8A—C5A	−1.42 (15)
C5A—C4A—C4—C3	−176.61 (14)	C9A—N9—C8A—C8	177.90 (14)
C5A—C4A—C4—C11	4.9 (2)	C10—N9—C8A—C5A	176.89 (14)
C9A—C4A—C4—C3	1.83 (19)	C10—N9—C8A—C8	−3.8 (2)
C9A—C4A—C4—C11	−176.69 (13)	C8A—N9—C9A—C1	−177.97 (14)
C4—C4A—C5A—C5	0.2 (3)	C8A—N9—C9A—C4A	0.79 (15)
C4—C4A—C5A—C8A	177.64 (14)	C10—N9—C9A—C1	3.7 (2)
C9A—C4A—C5A—C5	−178.42 (16)	C10—N9—C9A—C4A	−177.52 (14)
C9A—C4A—C5A—C8A	−0.96 (14)	N9—C9A—C1—C2	177.05 (14)
C4—C4A—C9A—N9	−178.70 (12)	C4A—C9A—C1—C2	−1.6 (2)
C4—C4A—C9A—C1	0.2 (2)		

### Hydrogen-bond geometry (Å, º)

Cg1, Cg2 and Cg3 are the centroids of rings N9/C8A/C5A/C4A/C9A, C1–C4/C4A/C9A, and C5/C5A/C8A/C8/C7/C6, respectively.

**Table:**

*D*—H···*A*	*D*—H	H···*A*	*D*···*A*	*D*—H···*A*
C8—H8···*Cg*1^i^	0.93	2.83	3.7091 (17)	159
C13—H13*A*···*Cg*2^ii^	0.97	2.91	3.6381 (17)	133
C14—H14*C*···*Cg*3^ii^	0.96	2.73	3.580 (2)	149

Symmetry codes: (i) *x*, −*y*−3/2, *z*−1/2; (ii) −*x*, *y*+1/2, −*z*+3/2.
